# Land degradation neutrality state and progress in the Central Region, Malawi

**DOI:** 10.1371/journal.pone.0362061

**Published:** 2026-11-05

**Authors:** Kennedy Sones Nazombe, Charles Bakolo, Brighton A. Chunga, Principal Mdolo, Jean Mtethiwa, Rodrick Mlewa, Kennedy Adamson, Devie Chilonga, Kelvin Chomanika

**Affiliations:** 1 Department of Land and Water Resources, Faculty of Life Sciences and Natural Resources, Lilongwe University of Agriculture and Natural Resources, Lilongwe, Malawi; 2 Ndata School of Climate and Earth Sciences, Malawi University of Science and Technology, Limbe, Malawi; 3 Ministry of Lands, Housing and Urban Development, Lilongwe, Malawi; 4 Department of Water and Sanitation, Faculty of Environmental Sciences, Mzuzu University, Mzuzu, Malawi; 5 Stockholm Environment Institute, World Agroforestry Centre, United Nations Avenue, Gigiri, Nairobi, Kenya; Griffith University, AUSTRALIA

## Abstract

Achieving Land Degradation Neutrality (LDN) is vital for the long-term sustainability of human well-being and the realisation of Sustainable Development Goal (SDG) 15.3.1. We estimated the overall indicator of SDG 15.3.1, the proportion of land that is degraded over the total land area in the Central Region, Malawi, from 2001 to 2022, using the Trends.Earth tool in QGIS. The study applied the United Nations Convention to Combat Desertification (UNCCD) Good Practice Guidance methodology for SDG Indicator 15.3.1 assessment, integrating land cover, land productivity, and soil organic carbon (SOC) sub-indicators. The default datasets in the Trends.Earth plugin were adopted for all sub-indicators. The study has revealed that: (i) The proportion of degraded land increased from 26.8% in the baseline period (2001–2015) to 41.2% in the monitoring period (2016–2022). Stable land area decreased from 62.2% to 55.7%, while improved land area declined from 10.0% to 2.0%. Overall, during the entire study period (2001–2022), 27.1% of the land remained degraded, 62.4% was stable, and 9.4% improved. (ii) Land with degraded productivity significantly declined from 74.5% to 29.3%. In contrast, land with stable productivity slightly increased from 1.1% to 1.9%. (iii) SOC levels remained largely stable (>97%), although slight losses occurred in cropland, grassland, and wetland areas. (iv) Land cover remained predominantly stable (96.7%) throughout the study period. The overall SDG 15.3.1 indicator indicates continued degradation trends exceeding the rate of land recovery and restoration. This study has highlighted key spatial patterns and identified insights into the priority areas for targeted LDN interventions in the Central Region of Malawi. The findings provide critical evidence for guiding sustainable land management strategies and accelerating progress toward achieving land degradation neutrality in Malawi.

## Introduction

Land degradation (LD) remains a critical environmental challenge of the century [1]. It has impacted the integrity of the ecosystem [2], agricultural productivity, and livelihoods [3]. Sub-Saharan Africa is recognised as a hotspot and the most exposed region to land degradation [4,5], as it accounts for over one-third of the global degraded land (about 715 hectares) [5]. In Malawi, LD hotspots constitute about 41% of the total landscape [6]. LD in Malawi is a result of several drivers, such as deforestation, unsustainable agricultural practices, soil erosion, and climate variability [4,6]. The effects of LD in Malawi are manifested in the decline of ecosystem services [7]. This has increased the vulnerability of communities that depend on natural resources. Addressing land degradation is essential for achieving sustainable development and improving regional food security.

The United Nations adopted the ambitious 17 Sustainable Development Goals (SDGs) to be achieved by 2030 in 2015 [8]. To track progress and measure the success of the SDGs and their 169 targets, a measurement framework with 232 indicators was established by the United Nations Statistical Commission [9]. Monitoring and reporting SDG progress is crucial for assessing policy effectiveness and identifying areas that need attention [10]. However, these indicators encounter challenges such as measurement difficulties and validation issues due to their subjective nature and the complexity of acquiring country-specific data [11]. Additionally, many indicators are not tailored to individual country contexts, making accurate progress assessment difficult [10].

SDG 15 (Life on land) is one of the key SDGs that directly relates to land management. The goal aims to halt, reduce, and reverse LD and achieve Land Degradation Neutrality (LDN) by 2030 [1]. LDN is “a state whereby the amount and quality of land resources necessary to support ecosystem function and services and enhance food security remain stable or increase within specified temporal and spatial scales and ecosystems” [12]. To achieve this goal, the SDG 15.3.1 indicator (UNCCD, 1994) was created to keep track of the “proportion of land that is degraded over the total land area” [13,14].

The United Nations Convention to Combat Desertification (UNCCD) introduced the concept of LDN to balance land degradation and restoration [15]. Land degradation is manifested in different forms, including water erosion, wind erosion, soil salinisation, and soil sealing [16]. No single metric can fully capture the overall health or state of the land due to the multifaceted nature of LD [17]. The LDN framework provides a scientific basis for monitoring land degradation trends and guiding sustainable land management interventions [18]. The two interconnected aspects of LDN are slowing down the rate at which non-degraded land deteriorates and speeding up the rate at which degraded land is restored [17]. To ensure consistency in the assessment of SDG 15.3.1, the UNCCD set the following three key biophysical indicators: land cover (to track changes in vegetation and land use patterns), land productivity dynamics (to assess changes in ecosystem productivity over time), and soil organic carbon (SOC) (to measure soil health and carbon sequestration capacity) [19]. The UNCCD recently released an addendum to the Good Practice Guidance for SDG Indicator 15.3.1 (Version 2.0), introducing the Status Matrix (3 × 3) approach (Teich et al. 2025). The Status Matrix compares current assessments with baseline conditions to classify land status as degraded (persistent degradation, recent degradation, and recent degradation), stable (baseline degradation, stability, and baseline improvement), or improved (recent improvement, recent improvement, and persistent improvement). This update strengthens the methodological framework for monitoring land degradation neutrality and enhances the interpretation of temporal changes in land conditions.

Global and regional efforts have been made to address land degradation and achieve the LDN state [1]. However, Malawi, like many other countries, still grapples with widespread land degradation [6]. Land degradation has severely affected agriculture, food security, and ecosystem health [20]. A weak industrial base with heavy reliance on agriculture characterises Malawi’s economy [21]. The Central Region is a major hub for maize, tobacco, and other cash crops in Malawi. However, increasing land pressure, poor land-use planning, and other factors have led to significant land degradation in the region. Achieving LDN in this region is critical for ensuring sustainable land productivity and food security. Despite the region’s significant role, land degradation trends and potential restoration strategies remain insufficiently studied. While the LDN framework and SDG 15.3.1 indicators provide a structured approach to monitoring LD, a lack of localised studies hinders effective implementation at the regional scale. No study has been performed in the central region of Malawi using the global UNCCD framework. This gap makes it difficult for decision-makers to prioritise degraded areas for intervention and monitor progress toward LDN targets. Therefore, this calls for systematic LDN assessments using geospatial techniques to provide data-driven insights into land degradation trends.

The study aimed to assess the LDN status of the central region of Malawi using the Trends.Earth Tool and the UNCCD LDN monitoring framework. It entailed quantification of land productivity, land cover, and soil organic carbon stock change from 2001 to 2022. We employed remote sensing and GIS-based analysis to quantify and monitor LD trends.

## Materials and methods

### Study area

The Central Region, Malawi, is located between 12°15′ S and 15°01′ S latitude and 32°48′ E and 36°02′ E longitude (Fig 1). The study area covers approximately 35,592 km², with most of the land dedicated to agriculture. The region houses the national capital city, Lilongwe. The region contains 9 districts, namely, Ntcheu, Salima, Ntchisi, Nkhotakota, Mchinji, Lilongwe, Kasungu, Dowa, and Dedza. According to the 2018 population and housing census, the region had a population of 7.5 million, which is about 43% of the national population [22]. The region experiences a cool-dry season from May to October and the hot-wet season from November to April. Most of the population in the region relies on rain-fed agriculture (Jørstad & Webersik, 2016). Direct drivers of land degradation in the region include poor farming practices, deforestation, overgrazing, urbanisation, uncontrolled bushfires, and natural disasters such as floods and droughts.

**Fig 1:**
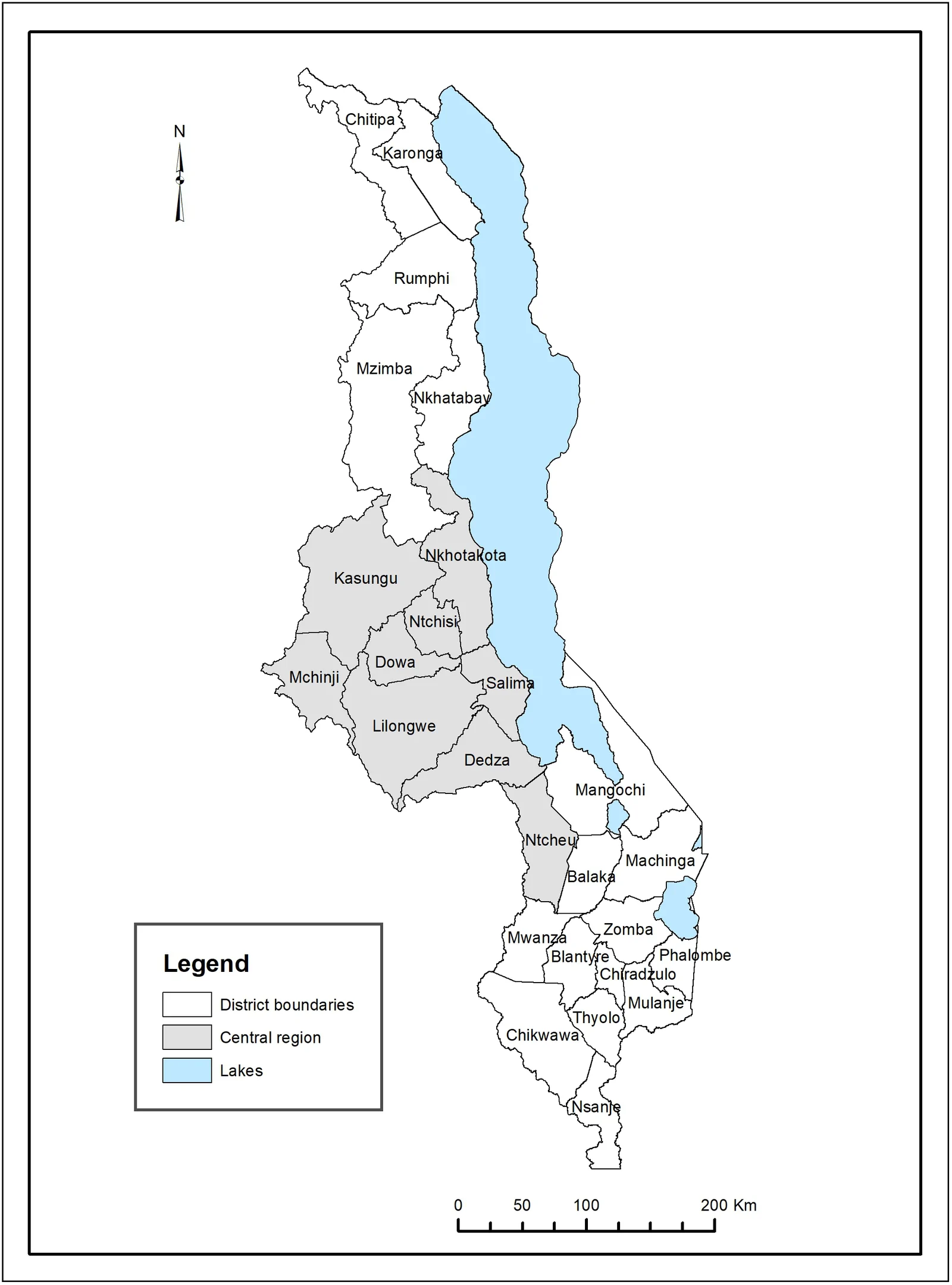
Map of Malawi showing the Central Region, the study area. Data source: Natural Earth (http://www.naturalearthdata.com/).

### Methodological framework

The study applied the UNCCD Good Practice Guidance methodology to assess SDG Indicator 15.3.1 [23] of the Central Region, Malawi. This was implemented in the Trends.Earth tool in QGIS. Trends.Earth integrates all analytical procedures required for the assessment of land degradation neutrality. It includes all preprocessing of input datasets, spatial analysis, and raster-based computations. The approach integrates the assessment of land cover, land productivity, and soil organic carbon (SOC) sub-indicators.

The UNCCD recommends a baseline period from 2000 to 2015 and a reporting period from 2016 onwards. In addition, the UNCCD recommends a default baseline period of 10–15 years (ending in 2015), depending on the availability of datasets (UNCCD 2016). We adopted the period from 2001 to 2015 as the baseline period, and 2016–2022 as the monitoring (or progress) period due to the data availability. Similarly, Cherif et al. (2023) used a period from 2001 to 2015 as a baseline to monitor LDN in Tunisia and Greece. The Trends. Earth tool computes the SDG Indicator 15.3.1 using the UNCCD framework. In addition, Trends.Earth uses datasets recommended by the UNCCD spatial databases as inputs for calculating land degradation indicators. The 2001–2015 period provided a benchmark for which progress towards achieving the SDG target 15.3 was assessed. The LDN state for the monitoring period was assessed based on the baseline, indicating changes experienced in terms of degraded land compared to the baseline period. The overall methodological approach is summarised in Fig 2.

**Fig 2:**
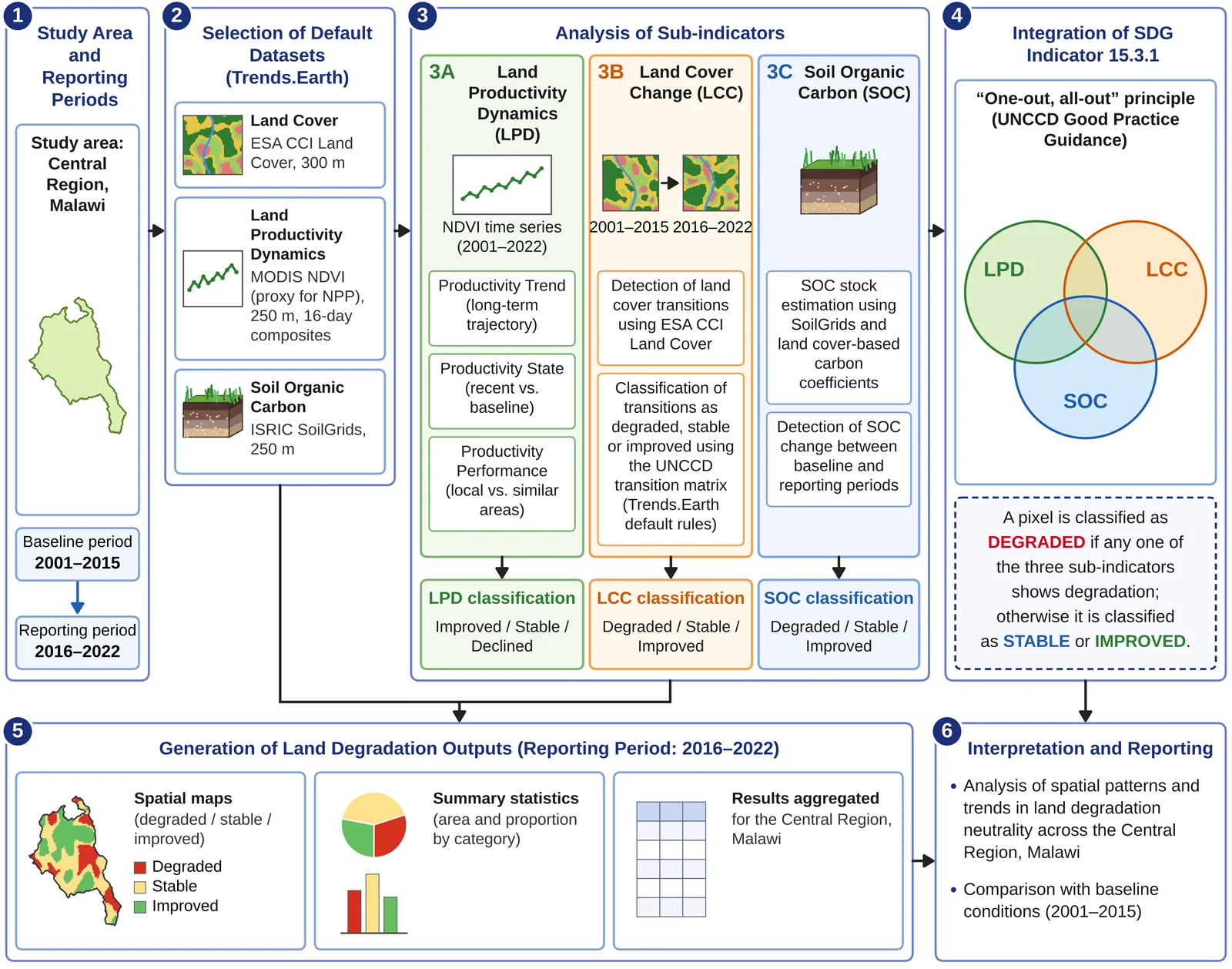
Methodological framework for assessing SDG 15.3.1 in the Central Region of Malawi. Source: authors.

### Data sources

We used free geospatial datasets available through the Trends.Earth Tool, an open-source platform for monitoring land degradation developed by Conservation International (http://trends.earth). The primary data sources included Land Productivity Dynamics (LPD), derived from Moderate Resolution Imaging Spectroradiometer (MODIS) Normalised Difference Vegetation Index (NDVI) time-series data, used to assess vegetation productivity trends. Land Cover Change (LCC) was evaluated based on the European Space Agency (ESA) Climate Change Initiative (CCI) land cover datasets. Soil Organic Carbon (SOC) Stocks –extracted from global soil databases (SoilGrids) were used to evaluate changes in soil carbon stocks. Finally, the administrative boundary shapefile layer was acquired from the Malawi Spatial Data Portal (MASDAP) (https://www.masdap.mw/) to define the study area.

### Data analysis and SDG indicator 15.3.1 calculation

#### Sub-indicator 1: Land productivity.

We utilised the Normalised Difference Vegetation Index (NDVI) from the Moderate Resolution Imaging Spectroradiometer (MODIS) to estimate Net Primary Productivity (NPP) in the Trends.Earth tool. The analysis followed the UNCCD good practice guidance for evaluating land productivity using three complementary metrics (trajectory/trend, state, and performance). The trajectory metric assesses the long-term trend in vegetation productivity over time using NDVI time series analysis. This identifies persistent increases or declines in productivity. The Pixel Residual Trend Analysis (RESTREND) approach was used to minimise the influence of precipitation variability on NDVI trends. RESTREND employs a linear regression model to predict NDVI based on a given amount of rainfall (Wessels et al. 2012). Productivity change that is not climate-induced is considered to be the difference between the predicted NDVI and the observed NDVI (the residual). MODIS global evapotranspiration product (MOD16A2) was used for the climate effect correction. While the Productivity State metric allows for the detection of recent productivity levels with historical baseline conditions. This helps to detect significant changes in ecosystem functioning between the baseline and reporting periods. Finally, the performance metric evaluates the relative productivity of a given pixel compared to ecologically similar areas with comparable climatic conditions and land cover characteristics. These three metrics were integrated within Trends.Earth to classify land productivity as degraded, stable, or improved for the SDG 15.3.1 assessment.

#### Sub-indicator 2: Land cover change.

To assess changes in land cover, we used the European Space Agency (ESA) Climate Change Initiative (CCI) land cover maps for the study area for the baseline and target years available in the Trends.Earth tool. We reclassified the land cover maps into forest/tree-covered, grassland, cropland, wetland, artificial area, bare land, and water. These are the 7 land cover classes required for reporting to the UNCCD. The description of the land cover types is provided in Table 1.

**Table 1:** Land cover classification description.

Code	Categories (UNCCD)	Classification description (ESA-CCI-LC)
1	Forest lands	Tree cover includes broadleaved or needle-leaved species, which may be evergreen or deciduous, with canopy density ranging from 15% to 50%, spanning from closed to open formations.
2	Grasslands	Areas with a mix of natural vegetation—such as trees, shrubs, and herbaceous plants—covering more than 50% of the land, combined with cropland occupying less than 50%. It also includes scrublands, grasslands, areas dominated by lichens and mosses, and sparsely vegetated zones where tree, shrub, or herbaceous cover is below 15%.
3	Croplands	This includes agricultural land (either rainfed or irrigated). This may or may not have herbaceous plants, trees, or shrubs, and can also be associated with patches of natural vegetation.
4	Wetlands	Areas with vegetative cover that are flooded by saline water; tree-covered areas inundated with fresh or brackish water; and regions with shrub or herbaceous vegetation submerged by fresh, saline, or brackish water.
5	Artificial	Urban or built-up areas.
6	Bare land	Bare areas/ exposed soil
7	Water bodies	Areas covered by water

#### Land cover accuracy assessment.

Congalton (1991) defined accuracy assessment as the degree to which remotely sensed data agrees with reference data. The reported overall accuracy of the ESA CCI Land Cover dataset at global ranges from 70% to 76% (Defourny et al. 2009; European Space Agency 2013). A preliminary accuracy assessment of the ESA CCI Land Cover dataset was conducted to ascertain the reliability of the maps for the study region. The Cochran equation was used to determine the sample size at a 95% confidence level and 5% margin of error. We computed the minimum required sample size to be 196 validation points (85% expected accuracy). However, a total of 500 stratified random points were generated for each land cover map (2001, 2015, 2016, and 2022). Confusion matrices were generated to compute the user´s accuracy, producer´s accuracy, overall accuracy, and kappa index. Generally, an overall accuracy of above 70% and a kappa index of above 0.60 is considered acceptable (Tan et al. 2021).

#### Land cover degradation assessment.

Based on the researcher’s familiarity with the local conditions and land degradation processes in the central region of Malawi, we identified land cover changes related to degradation. Fig 3 presents the transitions and indicates whether the changes represent degradation (–), improvement (+), or no change (0) in land condition.

**Fig 3:**
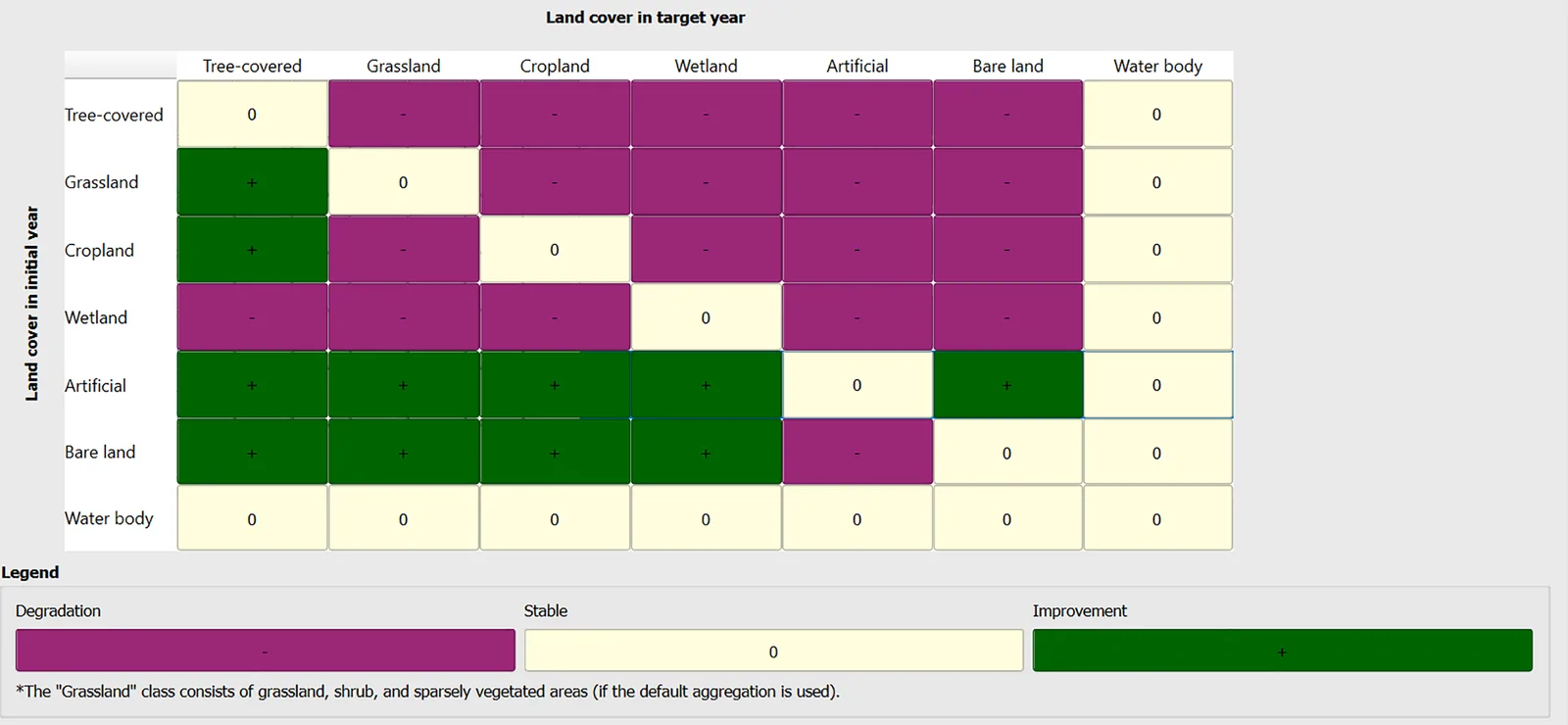
The defined effects of land cover change on land degradation in the study area. This figure is similar, but not identical, to the original image and is therefore for illustrative purposes only.

#### Sub-indicator 3: Soil organic carbon stocks.

To estimate the SOC of the study area, we used the default values in Trend.Earth. Trends.Earth employs a combined land cover/SOC method to assess changes in SOC and identify degraded areas. SOC reference values are derived from SoilGrids 250m for the top 30 cm of soil, though assigning a specific date to these values may introduce inaccuracies. Land cover maps are reclassified into seven UNCCD reporting classes (Table 1), requiring at least two time points for comparison. Changes in SOC stocks are estimated using land cover conversion coefficients recommended by the Intergovernmental Panel on Climate Change (IPCC) and UNCCD. This represents proportional SOC changes over 20 years, as spatially explicit data on land management and carbon inputs are often unavailable.

#### Overall SDG indicator 15.3.1 calculation.

The SDG Indicator 15.3.1 is determined by integrating three sub-indicators (land productivity, land cover change, and soil organic carbon stock) [19,23]. The trend component is derived through a spatio-temporal comparison of the three sub-indicators between the baseline and target periods. The Trends.Earth operates on the principle of “One-Out-All-Out” (1OAO), where all the sub-indicators are combined. If one of the sub-indicators shows degradation, the overall SDG indicator will show degradation. Following UNCCD’s LDN conceptual framework, a pixel was classified as degraded if at least one of the three indicators showed a negative trend. Similarly, when all the sub-indicators are stable, the final overall SDG indicator 15.3.1 will also indicate stability. The overall SDG indicator 15.3.1 reflects improvement when one or more of its sub-indicators are stable or improving, and none show signs of degradation. With Trend.Earth tool, no separate or independent statistical analyses were performed outside the Trends.Earth workflow. All computations were executed within a single integrated “one-click” processing framework, ensuring methodological consistency and reproducibility. For detailed technical information on the Trends.Earth tool and how it functions, refer to https://docs.trends.earth/en/latest/for_users/features/landdegradation.html.

## Results

### Land cover accuracy assessment

The preliminary results of the accuracy assessment of ESA CCI LC maps achieved moderate to high classification accuracies across the assessed years (Table 2). The overall accuracies of 88.6%, 78.6%, 82.8%, and 78.2% were achieved for the years 2001, 2015, 2016, and 2022, respectively. The Kappa Index ranged from 0.61 to 0.80, indicating substantial agreement between the classified maps and reference data.

**Table 2:** Overall accuracy, Kappa coefficient, user´s accuracy (UA), and producer´s accuracy (PA) for the ESA CCI LC maps of the Central Region, Malawi.

Land cover	2001		2015		2016		2022	
	UA (%)	PA (%)	UA (%)	PA (%)	UA (%)	PA (%)	UA (%)	PA (%)
Cropland	89.3	95.3	86.4	84.0	94.9	82.7	75.6	91.4
Tree covered	90.0	76.3	58.7	61.6	58.7	87.5	76.8	54.5
Grassland	70.0	63.6	60.0	75.0	70.0	70.0	80.0	64.0
Wetland	90.0	75.0	80.0	61.5	95.0	65.5	95.0	59.4
Artificial	85.0	94.4	60.0	85.7	70.0	87.5	85.0	89.5
Bareland	100.0	100.0						
Water	90.0	100.0	95.0	95.0	100.0	95.2	100.0	95.2
**Overall Accuracy**	**88.6**		**78.6**		**82.8**		**78.2**	
**Kappa Index**	**0.80**		**0.61**		**0.71**		**0.63**	

### The SDG 15.3.1 indicator for both the baseline and monitoring periods

The results indicate that 41.2% of the region was degraded in the baseline period (2001–2015), which is higher than the 26.8% recorded in the baseline period (Table 3). The improved land reduced from 10.0% in the baseline to 2.0% in the monitoring period. Similarly, land that remained stable declined from 62.2% in the baseline period to 55.7% in the monitoring period. The aggregated results for the whole period of study show that 62.4% remained stable, 9.4% improved, and 27.1% of the region was degraded. Fig 3 indicates that most of the land improvement was registered in the Nkhotakota Wildlife Reserve and the Kasungu National Park, as shown in Fig 4(c).

**Table 3:** The summary SDG indicator 15.3.1 during the baseline and monitoring periods in the Central Region, Malawi.

LD status	Baseline Period(2001–2015)		Monitoring Period(2016–2022)		Aggregated Change (2001–2022)	
	Area (ha)	Area (%)	Area (ha)	Area (%)	Area (ha)	Area (%)
Land area improved	368,933	10.0	74,348	2.0	349,173	9.4
Land area stable	2,300,111	62.2	2,060,695	55.7	2,307,736	62.4
Land area degraded	990,435	26.8	1,524,376	41.2	1,002,570	27.1
Land area with no data	38,862	1.1	38,922	1.1	38,862	1.1
Total land area	3,698,341	100.0	3,698,341	100.0	3,698,341	100.0

**Fig 4:**
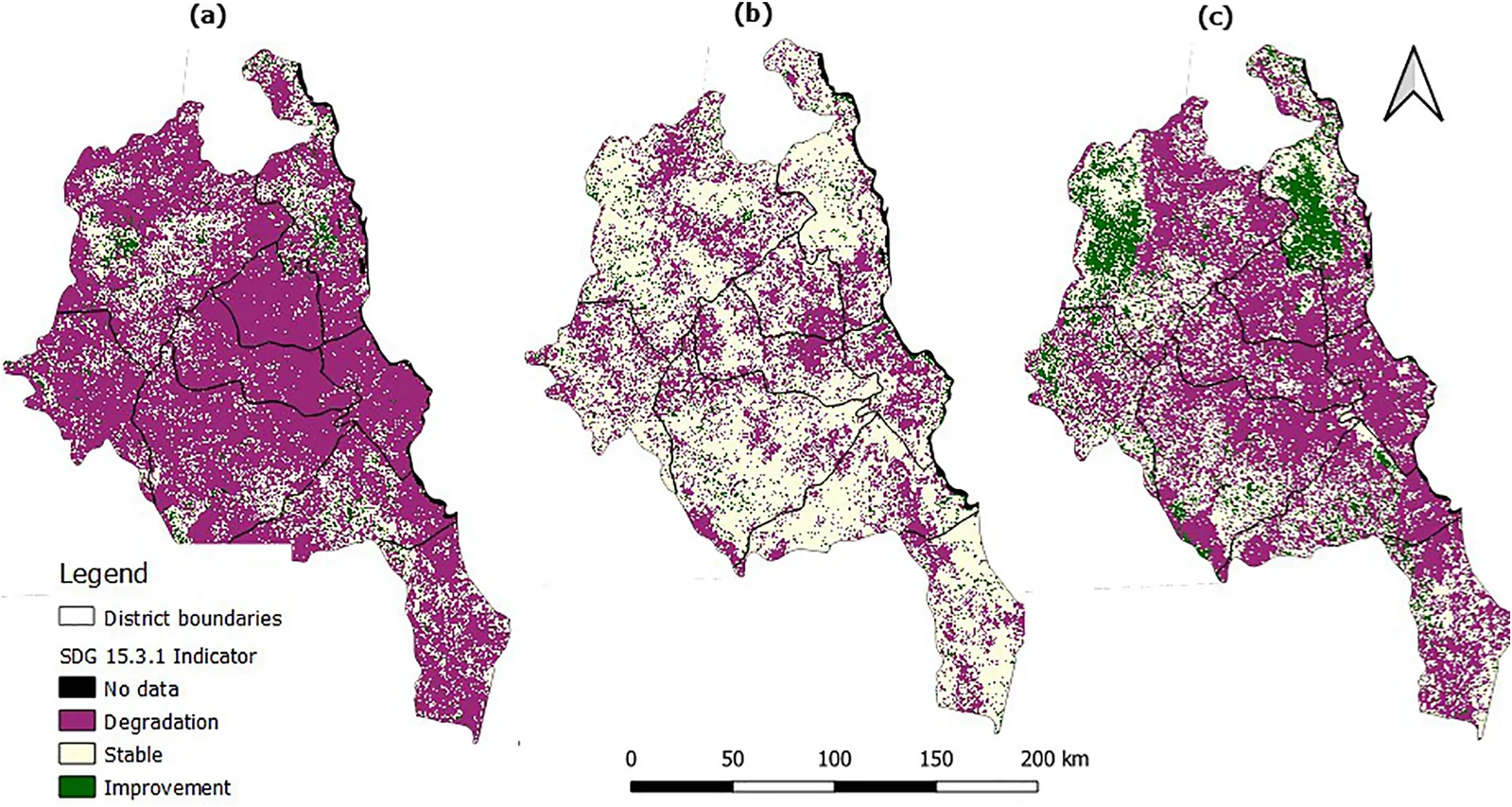
Spatial pattern of the SDG 15.3.1 indicator in the Central Region, Malawi, during the baseline period (a), the monitoring period (b), and the overall period (c). Source: Authors’ analysis using Trends.Earth was derived from the ESA Climate Change Initiative (CCI) Land Cover dataset, NASA MODIS-derived Land Productivity Dynamics data, and ISRIC–World Soil Information soil organic carbon data publicly available through Trends.Earth.

### Analysis of the SDG 15.3.1 Sub-Indicators

#### The land productivity sub-indicators.

The analysis of the land productivity trends indicates a significant improvement in stable productivity, increasing from 22.8% in the baseline period (2001–2015) to 56.3% in the monitoring period (2016–2022) (Table 4). Land with improved productivity slightly increased from 1.1% in the baseline to 1.9% in the monitoring period. Land with degraded productivity declined from 75.1% to 40.9%. The overall change (2001–2022) shows that 8.0% of the region recorded an improvement in land productivity, 64.9% remained stable, and 26.1% showed a decline in land productivity. Similarly, most of the land productivity improved in and around Kasungu National Park, Nkhotakota Wildlife Reserve, and other protected areas (Fig 5c).

**Table 4:** Land productivity indicators for the Central Region, Malawi, during the baseline and monitoring periods.

LD status	Baseline Period(2001–2015)		Monitoring Period(2016–2022)		Aggregated Change (2001–2022)	
	Area (ha)	Area (%)	Area (ha)	Area (%)	Area (ha)	Area (%)
Improved productivity	40,170	1.1	70,551	1.9	297,579	8.0
Stable productivity	843,867	22.8	2,080,786	56.3	2,400,308	64.9
Degraded productivity	2,778,243	75.1	1,510,943	40.9	964,393	26.1
No productivity data	36,061	1.0	36,061	1.0	36,061	1.0
Total land area	3,698,341	100.0	3,698,341	100.0	3,698,341	100.0

**Fig 5:**
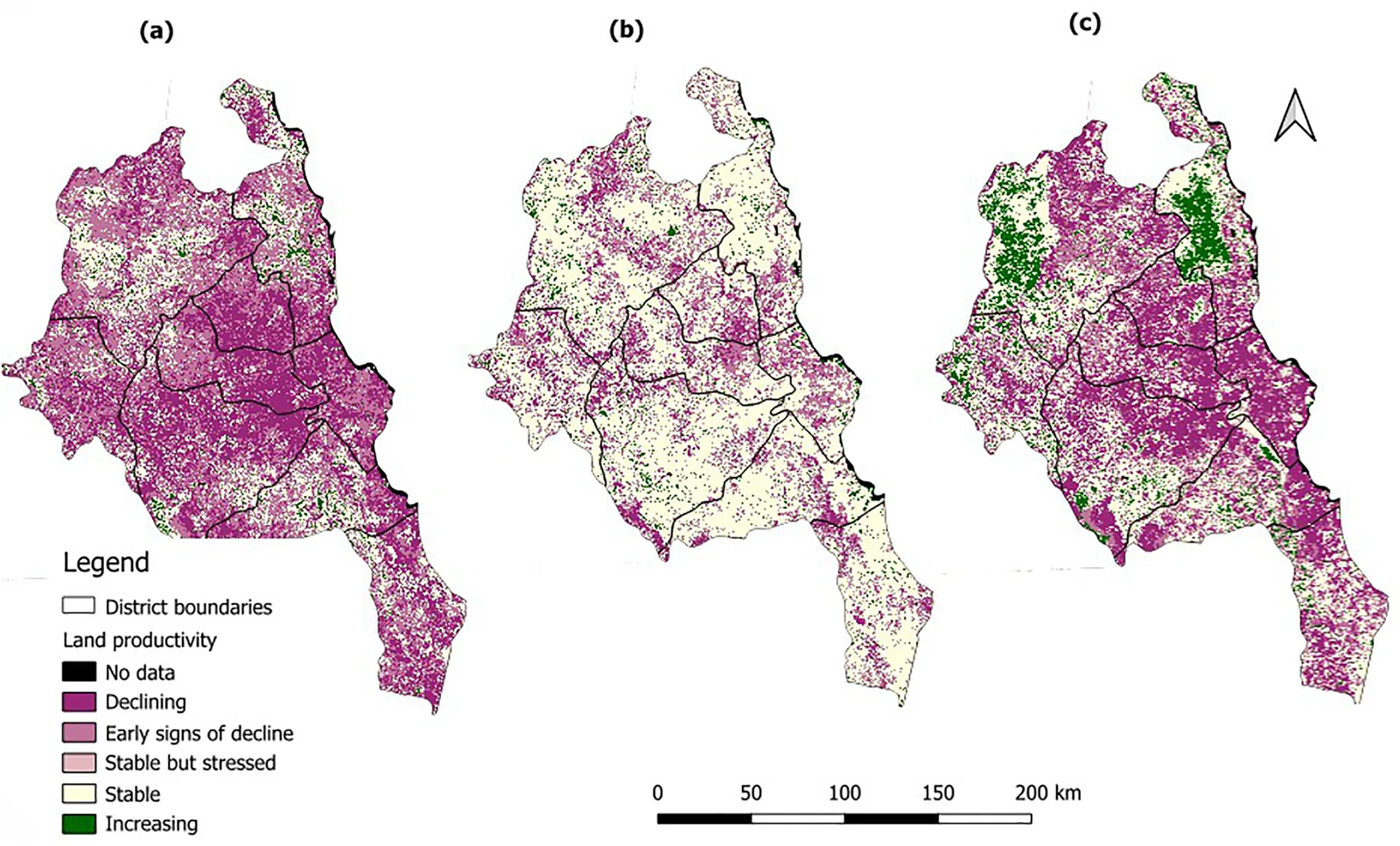
Spatial patterns of land productivity in Central Region, Malawi, during the baseline period (a), the monitoring period (b), and the overall period (c). Source: Authors’ analysis using Trends.Earth. Land productivity data were derived from publicly available NASA MODIS observations and processed as Land Productivity Dynamics indicators.

#### The soil organic carbon sub-indicators.

The SOC sub-indicators show that much of the study area remained stable (Table 5). The SOC stability slightly increased from 98.15% during the baseline period (2001–2015) to 99.61% in the monitoring period (2016–2022). At the same time, areas with improved SOC declined from 1.32% in the baseline period to 0.003% in the monitoring period. On the contrary, the land area with improved SOC increased by 1.57% over the entire period (2001–2022). Most of the SOC has been degraded in and around Lilongwe (Fig 6c).

**Table 5:** Soil Organic Carbon indicators for Central Region, Malawi, during the baseline, monitoring, and overall periods.

LD status	Baseline Period(2001–2015)		Monitoring Period(2016–2022)		Aggregated Change (2001–2022)	
	Area (ha)	Area (%)	Area (ha)	Area (%)	Area (ha)	Area (%)
Improved SOC	48,467	1.32	105	0.003	57,618	1.57
Stable SOC	3,593,830	98.15	3,646,824	99.607	3,570,722	97.51
Degraded SOC	18,208	0.50	13,500	0.369	32,165	0.88
No SOC data	1,228	0.03	786	0.021	1,228	0.03
Total land area	3,661,733	100.00	3,661,216	100.000	3,661,733	100.00

**Fig 6:**
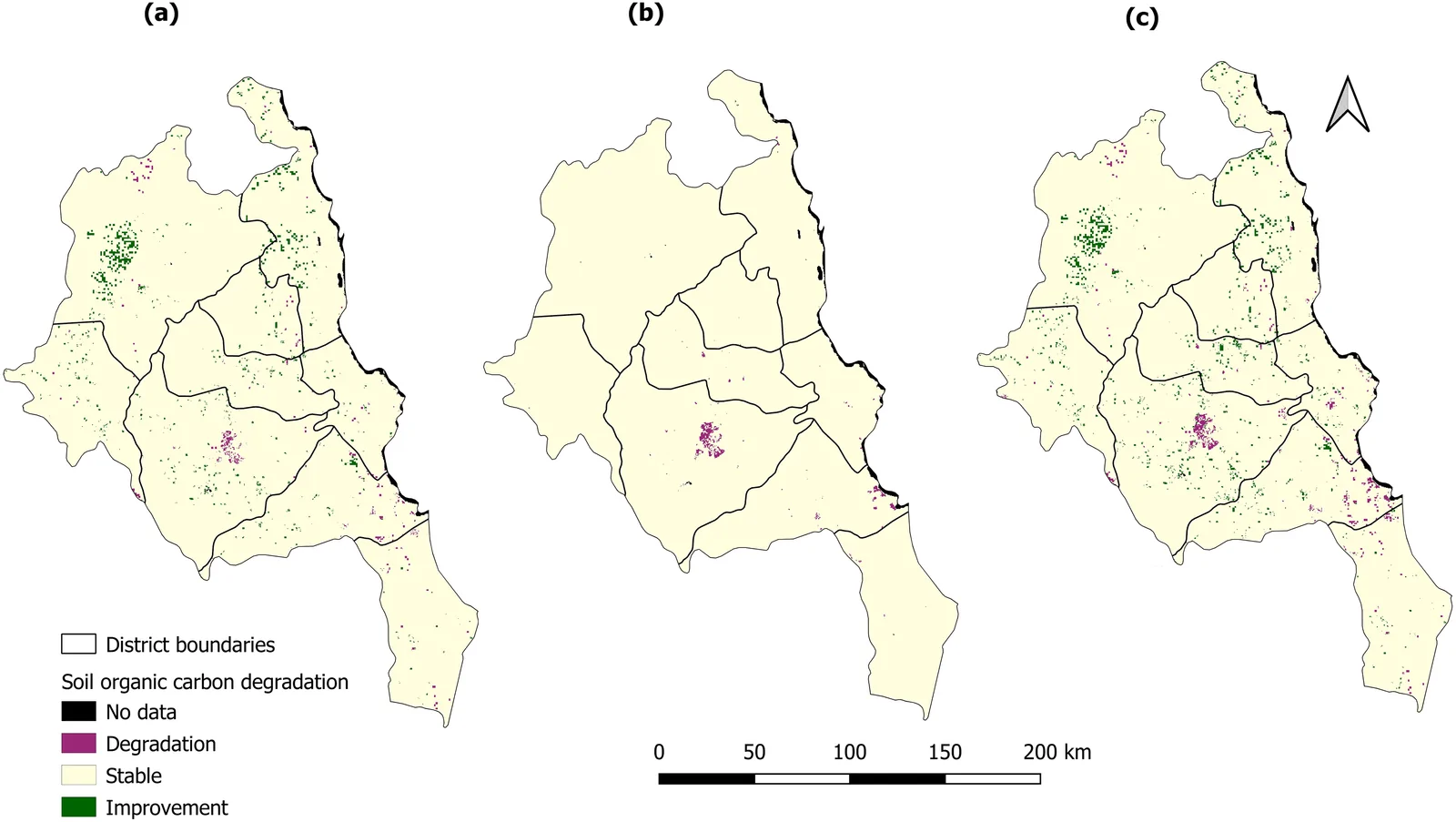
Spatial patterns of soil organic carbon distribution in Central Region, Malawi, during the baseline period (a), the monitoring period (b), and the overall period (c). Source: Authors’ analysis using Trends.Earth. Soil organic carbon data derived from publicly available SoilGrids developed by ISRIC–World Soil Information.

The distribution of SOC stocks indicates that the highest SOC was in cropland, followed by tree-covered areas in both the baseline and monitoring periods (Table 6). At the same time, wetlands contained the highest SOC of 70 tC/ha. Overall, the study revealed slight changes in SOC (tC/ha). SOC in artificial land cover areas decreased from 44 tC/ha to 37 tC/ha from the baseline period to the monitoring period. In contrast, SOC content in tree-covered areas slightly increased from 65 tC/ha in the baseline period to 66 tC/ha in the monitoring period. SOC content in grasslands, croplands, and wetlands remained constant. In addition, tree-covered areas registered a 2.3 million-tonne increase in SOC content, representing 4.5%. SOC decreased in grasslands, croplands, and wetlands by 0.2, 2, and 0.1 million tonnes, respectively, between the baseline and monitoring periods.

**Table 6:** Changes in soil organic carbon stocks for Central Region, Malawi, during the baseline and monitoring periods.

Land cover class	SOC (tC/ha)		SOC Stocks (Million tC)		Change in SOC Stocks	
	Baseline	Monitoring	Baseline	Monitoring	(Million tC)	%
Tree-cover	65	66	51.3	53.6	2.3	4.5
Grassland	63	63	8.4	8.2	−0.2	−2.4
Cropland	55	55	146.5	144.5	−2.0	−1.4
Wetland	70	70	5.7	5.6	−0.1	−1.8
Artificial	44	37	0.6	0.8	0.2	33.0
Bare land	0.0	0.0	0.0	0.0	0.0	0.0

#### The land cover sub-indicators.

The analysis of the land cover degradation shows that the land cover is dominantly stable in both baseline and monitoring periods (Table 7). The land cover stability increased from 97.7% during the baseline period (2001–2015) to 99% in the monitoring period (2016–2022). In contrast, 96.7% of the land cover remained stable for the aggregated period (2001–2022). However, areas with improved land cover declined from 1.2% in the baseline period to 0.2% in the monitoring period. The land area with improved land cover increased to 1.6% over the entire period.

**Table 7:** Land cover indicators for the Central Region, Malawi, during the baseline, monitoring, and overall periods.

LD status	Baseline Period(2001–2015)		Monitoring Period(2016–2022)		Aggregated Change (as of 2022)	
	Area (ha)	Area (%)	Area (ha)	Area (%)	Area (ha)	Area (%)
Improved land cover	44,531	1.2	8,634	0.2	59,676	1.6
Stable land cover	3,611,606	97.7	3,672,858	99.3	3,577,386	96.7
Degraded land cover	42,203	1.1	16,849	0.5	61,279	1.7
No data for land cover	0	0.0	0.0	0.0	0.0	0.0
Total land area	3,698,341	100.0	3,698,341	100.0	3,698,341	100.0

The land cover spatial and temporal distribution of the seven classes shows significant changes (Table 8 and Fig 7). The region is dominated by croplands covering 73.3% of the area in 2001 and 72.2% in 2022. The dominance of croplands is followed by tree-covered areas, constituting 19.52% in 2001 and 20.6% in 2022 of the study area. From 2001 to 2022, tree-covered, artificial, and waterbody land cover types increased by 5.3%, 340.9%, and 1.2%, respectively. In contrast, grassland, cropland, and wetlands declined by 9.3%, 1.53%, and 0.2%, respectively.

**Table 8:** Land cover distribution and change in the Central Region, Malawi.

Land cover class	2001		2022		Change (2001–2022)	
	ha	%	Ha	%	ha	%
Tree-covered	721,944	19.52	760,439	20.56	38,495	5.33
Grassland	142,743	3.86	129,525	3.50	−13,219	−9.26
Cropland	2,712,407	73.34	2,670,883	72.22	−41,523	−1.53
Wetland	79,939	2.16	79,747	2.16	−192	−0.24
Artificial	4,693	0.13	20,690	0.56	15,997	340.88
Bare land	7	0.00	0	0.00	−7	−100.00
Water body	36,608	0.99	37,057	1.00	449	1.23
Total	3,698,341	100.00	3,698,341	100.00		

**Fig 7:**
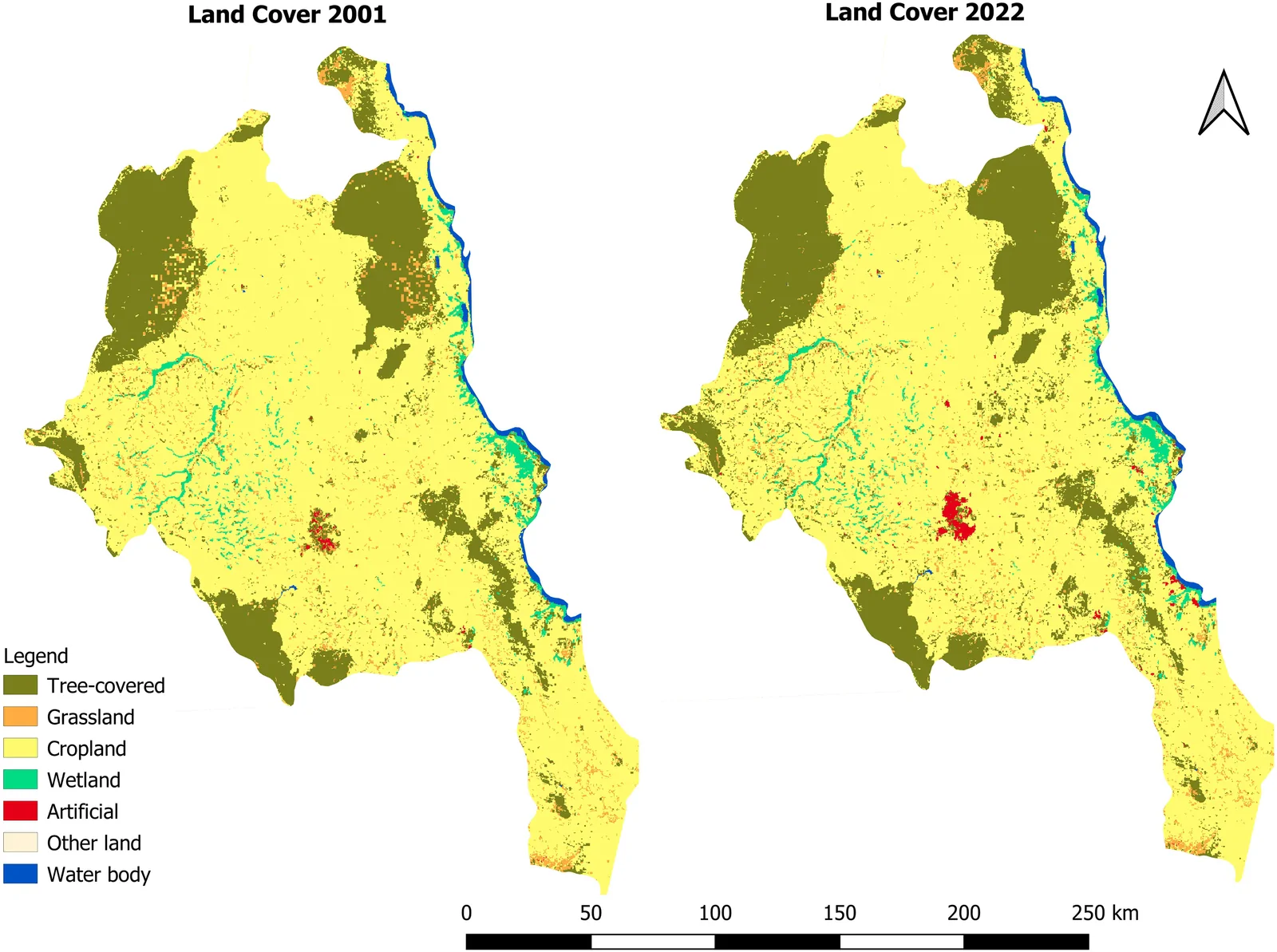
Spatial patterns of land cover distribution in Central Region, Malawi, in 2001 and 2022. Source: Authors’ analysis using Trends.Earth. Land cover data were derived from the publicly available ESA Climate Change Initiative (CCI) Land Cover dataset.

The land cover transition matrix (Table 9) indicates the gains and losses in each land cover class. The gains in tree-covered areas from 2001 to 2022 were mainly from croplands (37,550 ha) and grasslands (22,126 ha). At the same time, artificial areas expanded at the expense of tree cover, grassland, and cropland. Cropland mainly gained from tree cover (18,071 ha) and grassland (4,420 ha) and experienced a net loss of 64,425 ha.

**Table 9:** Land cover transition matrix for Central Region, Malawi, from 2001 to 2022.

	2022									
	Land cover	Trees	Grassland	Cropland	Wetland	Artificial	Bare land	Water	Total	Losses
**2001**	Trees	698,218	2,330	18,071	2,749	224	0	352	721,944	23,726
	Grassland	22,126	112,560	4,420	8	3,556	0	75	142,743	30,184
	Cropland	37,550	14,635	2,647,981	128	12,030	0	82	2,712,407	64,425
	Wetland	2,545	0	412	76,787	164	0	30	79,939	3,151
	Artificial	0	0	0	0	4,693	0	0	4,693	0
	Bare land	0	0	0	0	7	0	0	7	0
	Water	0	0	0	75	15	0	36,518	36,608	90
	Total	760,439	129,525	2,670,883	79,747	20,690	0	37,057	3,698,341	
	Gains	62,221	16,965	22,902	2,959	15,997	0	539		

## Discussion

### Trends in SDG 15.3.1 indicator and land degradation neutrality

In this study, we assessed the LDN in the Central Region of Malawi. The study has shown that the largest proportion of the region remained stable. However, the stability declined from 62.2% in the baseline period to 55.7% in the monitoring period. In addition, the proportion of degraded land increased from 26.8% in the baseline period to 41.2% in the monitoring period. Improved land declined from 10.0% in the baseline period to only 2.0% in the monitoring period. The overall SDG 15.3.1 indicator shows a trend towards degradation. This shift demonstrates that land degradation processes intensified between 2016 and 2022, exceeding the rate of land recovery and restoration. An increase in degradation may be attributed to several interacting factors, including population growth, loss of grasslands, and an increase in built-up areas. The population of the region has been increasing steadily over time, rising from 3 million in 1987–4 million in 1998, 5.5 million in 2008, and reaching 7.5 million in 2018 [22]. Despite that, the improvements in land management practices in the study area are insufficient to offset degradation processes; some recent improvements were noted. This is particularly observed in conservation areas such as Nkhotakota Wildlife Reserve and Kasungu National Park. This is in line with the provisions of the National Forest Landscape Restoration Strategy [24]. This trend supports global attempts to reduce land degradation through afforestation, reforestation, and sustainable land management practices.

In the Kiteto and Kongwa districts of Tanzania, contrary trends were reported. A decline in the proportion of degraded land from 70% in the baseline period to 16% during the monitoring period [25]. Similarly, the proportion of degraded land declined from 9.97% in the baseline period to 6.26% in the reporting period in Tunisia [26]. In Botswana, an estimated 32.6% of land was degraded between 2000 and 2015 based on customised datasets, whereas global datasets indicated a higher estimate of 51.4% [27].

### Land productivity dynamics

The present study has recorded significant progress in land productivity trends, as evidenced by an increase in land with stable productivity from 22.8% in the baseline period to 56.3% in the monitoring period. Land with degraded productivity decreased from 75.1% in the baseline period to 40.9% in the monitoring period. Land productivity slightly increased from the baseline period (1.1%) to the reporting period (1.9%). For the overall period (2001–2022), land productivity increased by 8.0%. Generally, the land productivity sub-indicator shows signs of slight recovery and increased stabilisation during the monitoring period compared to the baseline period. The increase in land cover types with high productivity, such as tree cover, may be attributed to this. The observed improvements in Kasungu National Park and Nkhotakota Wildlife Reserve justify the use of protected areas as an effective conservation strategy for maintaining and restoring ecosystem functions. Further analysis indicates that both protected areas are under co-management with surrounding communities, showing the central role of community involvement in achieving ecosystem restoration and sustainable land management [24]. Globally, similar trends have been reported, with land in protected areas gaining more productivity than land outside protected areas [28]. The study has further shown that 26.1% of the study area still experienced productivity loss between 2001 and 2022. Continued investments in land restoration beyond protected areas, where land use pressures may be higher, need to be prioritised to achieve LDN status. Declining land productivity directly affects crop yields, thereby putting food security and livelihoods at risk, especially in low-income countries [29]. Similarly, land with degraded productivity increased from 8.2% in the baseline period to 12.2% in the monitoring period in the semiarid region of Tanzania; land with stable productivity declined from 91.3% to 87.7% [25]. On the contrary, land with degraded productivity in Tunisia declined from 8.7% to 6.2% in the baseline (2001–2015) and monitoring (2016–2019) periods, respectively [26].

### Soil organic carbon trends

The assessment of SOC stocks showed that much of the region experienced stable SOC levels. Only less than 1% of the region was classified as degraded in both the baseline and monitoring periods. The stability may be attributed to the stability in cropland, which contained the highest stocks of SOC. The higher stock of SOC in the region is because cropland covers most of the area (about 70%). However, due to unsustainable agricultural systems and vegetative cover, croplands tend to have lower stocks of SOC [13,30]. While the overall SOC stocks remained stable, there were notable changes across land cover types. Tree-covered areas showed an increase in SOC, while grassland, cropland, and wetlands showed slight declines. The recorded losses in SOC in cropland (2.0 million tC), wetlands (0.1 million tC), and grassland (0.2 million tC) indicate the need for sustainable agricultural practices to mitigate soil carbon depletion. Slight variations in SOC have even the potential to significantly influence the carbon cycle and atmospheric CO_2_ concentrations, with important consequences for natural ecosystems and biodiversity [31,32]. Similar trends were observed in the Matabeleland North Province of Zimbabwe, where low SOC levels in croplands were recorded, and much of the region (59.55%) maintained stable carbon stocks [33]. The Semiarid Region, Tanzania, experienced overall stable SOC stocks using UNCCD default datasets. However, slight losses in SOC were observed in grassland, wetlands, and forest land cover [25].

### Land cover changes and their impact on land degradation

We analysed land use dynamics in the Central Region, Malawi, using the European Space Agency Climate Change Initiative (ESA-CCI) datasets to assess land degradation changes. The results indicated that the region’s landscape changed significantly between 2001 and 2022. An increase in tree cover was observed in the protected areas, such as Kasungu National Park and Nkhotakota Wildlife Reserve. The tree cover gained from croplands and grasslands by 37,550 ha and by 22,126 ha, respectively. The gains in forest cover may be linked to the land restoration efforts in the protected areas. An increase in tree cover is a positive step towards achieving Malawi’s LDN voluntary commitment of reestablishing 820,000 hectares of degraded native forest by the year 2030 [34]. This also speaks directly to Malawi’s related commitment of restoring 4.5 million hectares of degraded landscapes by the same year 2030 [24]. Gains in forest cover from grassland and cropland were also reported in Zimbabwe; however, deforestation for farming continued in the same period [33]. The study also recorded a significant expansion of artificial/built-up areas by 340.9% from 2001 to 2022. The built-up areas expanded into forests, cropland, and grasslands. The expansion of artificial areas at the expense of natural land cover classes could have long-term ecological implications. This includes reduced biodiversity and increased land fragmentation [35] against the provisions of the country’s National Biodiversity Strategy and Action Plan II [36]. It is projected that 52% of terrestrial species will be lost due to the expansion of urban areas by 2050 globally [37]. In addition, grassland and wetlands declined by 9.3% and 0.2%, respectively. In a national-level study, grassland in Malawi increased by 52%, while wetlands declined by 27.6% between 2001 and 2022 [7]. The continued loss of wetlands raises concerns about whether Malawi has met its voluntary target of halting the conversion of wetlands to other land uses by 2020. Grassland and wetland loss have implications for the ecosystem services provided by them, such as habitat provision, carbon sequestration, flood control, and water purification [38]. In a similar study in Matabeleland Province in Zimbabwe, settlements expanded by 163% from 1992 to 2020 [33]. In contrast, settlements declined in the Upper Blue Basin, Ethiopia, during the assessment of the LDN status of the Rib watershed [39]. In the North Wello Zone, Ethiopia, built-up areas expanded by 270.7% from 2000 to 2023 [40]. A further analysis of the land cover degradation status reveals that the proportion of land with stable land cover increased from 97.7% in the baseline period to 99.3% in the monitoring period. The land cover stability suggests that major land cover transformations have been limited. Land cover change stability is essential for maintaining ecological balance. In addition, 1.6% of the region recorded improvement in land cover. This demonstrates slow but positive progress in ecosystem restoration.

### Implications for sustainable land management and policy

Land degradation has serious environmental consequences for water quality, biodiversity, and ecosystem services [41]. The cost of land degradation on agriculture at the global scale is estimated at US$500 billion/yr. The observed land degradation in the study area suggests that sustainable land management strategies and restoration efforts are minimal in the region [24]. The decline in land stability and land improvement indicates insufficient implementation of land restoration practices in the region. In line with this, Malawi set its voluntary targets for achieving LDN, which include enhancing the productivity of 754,320 hectares of cropland and restoring 4.5 million hectares of degraded landscapes by 2030 [24,34]. Increasing SOC stocks from 44.7 tonnes/ha (in 2015) to 55 tonnes/ha (by 2025) in cropland [34]. By 2030, Malawi also seeks to restore one million hectares of degraded land for agriculture [34]. Land use planning based on the ecosystem approach has been identified as a key strategy for achieving LDN [42]. This promotes sustainable land management practices to prevent natural ecosystems from degradation through rehabilitation, reclamation, and renaturalisation [43]. Additionally, strengthening the existing legal policy and legal framework is crucial for achieving LDN status. Malawi’s legislation on LDN has been characterised as having weak avoid, reduce, and reverse priorities [44]. Currently, there is no clear legal responsibility for land users to limit actions that are contrary to achieving LDN [44]. Lastly, engaging local communities in land management efforts and strengthening policies that promote sustainable land use are important to attain Malawi’s LDN targets. The success stories registered in the featured protected areas of Kasungu National Park and Nkhotakota Wildlife Reserve (among other forest reserves in the country) support this recommendation [45].

### Limitations of the study

One of the limitations of this study is the use of global remote sensing datasets with medium spatial resolution (250 m to 300 m) as inputs to Trends.Earth. The 300 m spatial resolution of the ESA CCI Land Cover dataset used in this study may limit the detection of small-scale land cover changes and fragmented landscape features. Despite the recommendation to use high-quality national datasets, the national land cover datasets are not available to fully cover the temporal coverage requirement in Malawi. Currently, the national reporting to the UNCCD uses default global datasets only (Tier 1 Reporting) due to the unavailability of data for Malawi. Secondly, inaccuracies in the ESA CCI Land Cover dataset. A preliminary accuracy assessment of the ESA CCI Land Cover dataset for Malawi showed moderate to high classification performance across the assessed years (overall accuracies ranging from 78.2% to 88.6%). However, tree-covered areas, grasslands, and wetlands showed comparatively lower producers’ and users’ accuracies in some years. This suggests possible confusion among spectrally similar classes and omission errors. Therefore, some uncertainty may remain in the land cover change analysis and subsequent SDG 15.3.1 estimates. Another limitation is the inherent uncertainties in the calculation of the SOC sub-indicator, which relies on the SoilGrids datasets. The SoilGrids dataset exhibits variability and uncertainty in SOC estimates across different regions. Under and overestimation of carbon stocks were in the USA, France, England, and Wales [46]. In addition, SOC stock changes in Trends.Earth are inferred from land cover transitions, which may introduce further uncertainty into the assessment. Lastly, direct field-based validation of the final SDG 15.3.1 outputs was not possible because of the absence of ground reference data for the study area. Consequently, the interpretation of degradation patterns relied on known environmental conditions in Malawi. Nevertheless, the use of globally consistent datasets and harmonised methods implemented through tools such as Trends.Earth, offers a reproducible, consistent, and transparent framework for assessing land degradation.

## Conclusion and future recommendations

In this study, we assessed the status and progress in achieving LDN in the Central Region, Malawi. In addition, the three sub-indicators of LDN (land cover, land productivity, and SOC trends) were assessed. The study has shown that the proportions of improved, stable, and degraded land for the overall SDG 15.3.1 indicator during the baseline period were 10.0%, 62.2%, and 26.8%, respectively. In the monitoring period, the proportion of improved land declined to 2.0%, land stability decreased to 55.7%, and the proportion of degraded land increased to 41.2%. The overall SDG 15.3.1 indicator shows a trend towards continued degradation despite the largest proportion of the region remaining stable (62.4%). An assessment of the LDN sub-indicator on land productivity trends indicates an increase in land productivity by 8.0% from 2001 to 2022, signifying the impact of land restoration initiatives being implemented in the region. In addition, the evaluation of the SOC levels indicator shows high stability in SOC stocks. However, slight losses in SOC stocks were noted in grassland, cropland, and wetland land cover types. Therefore, targeted interventions in grassland, cropland, and wetlands are essential to improve SOC stocks. The proportion of land with improved land cover declined from 1.2% in the baseline period to 0.2%. The proportion of land with improved land cover declined from 1.2% in the baseline period to 0.2% in the monitoring period. However, the proportion of land with degraded land cover declined from 1.1% to 0.5%. This represents partial progress toward LDN under the land cover sub-indicator. The overall findings suggest that land degradation processes are progressing at a faster rate than restoration efforts in the region.

The findings of this study have policy implications for guiding land management practices and aligning local actions with both national and global LDN aspirations. However, the study is limited by the reliance on default global datasets from the Trends.Earth tool. Global datasets suffer from the drawback of not capturing fine-scale land changes, including local land management practices. Therefore, future research should incorporate localised datasets to refine degradation assessments and enhance decision-making. The use of global datasets aligns with international standards such as those recommended by the UNCCD (global LDN reporting frameworks) to ensure standardisation and comparability of studies across regions and countries. In addition, future studies should include field observations and ground reference datasets to validate degradation patterns and reduce uncertainties associated with remote sensing products. In conclusion, the study highlights the state of land degradation and the challenges that persist in the region. To attain LDN status by 2030, a range of activities is required to develop an enabling environment and to upscale sustainable land management practices in the region.
